# A network meta-analysis and systematic review of change in QRS duration after left bundle branch pacing, His bundle pacing, biventricular pacing, or right ventricular pacing in patients requiring permanent pacemaker

**DOI:** 10.1038/s41598-021-91610-8

**Published:** 2021-06-09

**Authors:** Nithi Tokavanich, Narut Prasitlumkum, Wimwipa Mongkonsritragoon, Wisit Cheungpasitporn, Charat Thongprayoon, Saraschandra Vallabhajosyula, Ronpichai Chokesuwattanaskul

**Affiliations:** 1 Division of Cardiovascular Medicine, Department of Medicine, Cardiac Center, Faculty of Medicine, Chulalongkorn University and King Chulalongkorn Memorial Hospital, Thai Red Cross Society, Bangkok, Thailand; 2 Division of Cardiology, University of California Riverside, Riverside, CA USA; 3 Department of Pediatrics, Faculty of Medicine, Chulalongkorn University and King Chulalongkorn Memorial Hospital, Thai Red Cross Society, Bangkok, Thailand; 4 Division of Nephrology and Hypertension, Mayo Clinic, Rochester, MN USA; 5 Section of Interventional Cardiology, Division of Cardiovascular Medicine, Department of Medicine, Emory University School of Medicine, Atlanta, GA USA

**Keywords:** Cardiology, Cardiac device therapy

## Abstract

Cardiac dyssynchrony is the proposed mechanism for pacemaker-induced cardiomyopathy, which can be prevented by biventricular pacing. Left bundle branch pacing and His bundle pacing are novel interventions that imitate the natural conduction of the heart with, theoretically, less interventricular dyssynchrony. One of the surrogate markers of interventricular synchrony is QRS duration. Our study aimed to compare the change of QRS duration before and after implantation between types of cardiac implantable electronic devices (CIEDs): left bundle branch pacing versus His bundle pacing versus biventricular pacing and conventional right ventricular pacing. A literature search for studies that reported an interval change of QRS duration after CIED implantation was conducted utilizing the MEDLINE, EMBASE, and Cochrane databases. All relevant works from database inception through November 2020 were included in this analysis. A random-effects model, Bayesian network meta-analysis was used to analyze QRS duration changes (eg, electrical cardiac synchronization) across different CIED implantations. The mean study sample size, from 14 included studies, was 185 subjects. The search found 707 articles. After exclusions, 14 articles remained with 2,054 patients. The His bundle pacing intervention resulted in the most dramatic decline in QRS duration (mean difference, − 53 ms; 95% CI − 67, − 39), followed by left bundle branch pacing (mean difference, − 46 ms; 95% CI − 60, − 33), and biventricular pacing (mean difference, − 19 ms; 95% CI − 37, − 1.8), when compared to conventional right ventricle apical pacing. When compared between LBBP and HBP, showed no statistically significant wider QRS duration in LBBP with mean different 6.5 ms. (95% CI − 6.7, 21). Our network meta-analysis found that physiologic pacing has the greatest effect on QRS duration after implantation. Thus, HBP and LBBP showed no significant difference between QRS duration after implantation. Physiologic pacing interventions result in improved electrocardiography markers of cardiac synchrony, narrower QRS duration, and might lower electromechanical dyssynchrony.

## Introduction

Pacemaker-induced cardiomyopathy (PCM) is defined as a fall in left ventricular ejection fraction (LVEF) of more than 10% from the baseline after other differential diagnoses are excluded^1^. More than 20% of right ventricular (RV) pacing has been found to be highly associated with an increased incidence of heart failure^2^. The prevalence of PCM has been reported to be up to 9% in chronic RV pacing patients^3^.

The key pathophysiology in PCM is the hemodynamic effect of pacing-induced cardiac dyssynchrony. RV pacing results in delayed activation of left ventricular (LV) cardiac muscle cells, causing abnormal contraction and a negative inotropic effect in mammals^4,5^. This phenomenon has also been confirmed by histologic changes in cardiac muscle cells, in which myofibril disarrays have been observed^6^.

The main objective of PCM therapy is to restore cardiac ventricular synchrony. The standard treatment is to upgrade from conventional RV pacing to biventricular pacing (BiV), so-called cardiac resynchronized therapy (CRT), by adding an LV epicardial lead. CRT is associated with lower mortality, fewer urgent care visits for acute heart failure, and improved LV end-systolic volume index^7^. Other methods of resynchronization are His bundle pacing (HBP) and left bundle branch pacing (LBBP), also called physiologic pacing^8,9^. These techniques differ in the success rates of implantation and clinical outcomes across studies; however, there have been no studies comparing the benefits and effects of these interventions. In the present study, we aim to investigate the effect of these different pacing techniques on cardiac synchronization compared to BiV and conventional RV pacing.

## Methods

### Literature review and search strategy

The protocol for this network meta-analysis is registered with PROSPERO (International Prospective Register of Systematic Reviews; no. CRD 42020210277) ^10^. A systematic literature search of MEDLINE (1946 to November 2020), EMBASE (1988 to November 2020), and the Cochrane Database of Systematic Reviews (1993 to November 2020) was conducted to compare the following outcomes: electromechanical dyssynchrony, as represented by QRS duration, following cardiac implantable electronic device (CIED) implantation between HBP, LBBP, BiV, and conventional RV apical pacing treatments.

The systematic literature review was undertaken independently by 2 investigators (R.C. and N.T.) applying a search approach that incorporated the terms of “His bundle pacing,” “Left bundle branch pacing,” “Biventricular pacing,” “Cardiac resynchronization therapy,” and “Right ventricular pacing,” alone and in combination. The results of this search are provided in Supplemental Data 1. A manual search for conceivably relevant studies was also performed using references of the included articles. No language limitation was applied. This study was conducted in accordance with the Strengthening the Reporting of Observational Studies in Epidemiology (STROBE)^11^ and the Preferred Reporting Items for Systematic Reviews and Meta-Analysis (PRISMA) statements^12^.

### Selection criteria

Data from observational studies (cohort, case–control, or cross-sectional studies) and randomized studies were used for this analysis. Eligible studies included those that provided data on the clinical characteristics, type of CIEDs, and QRS duration prior to and after device implantation. Inclusion was not limited by study size. Retrieved articles were individually reviewed for their eligibility by 2 researchers (R.C. and N.T.). Discrepancies were discussed and resolved by a third researcher (N.P.). The Newcastle–Ottawa quality assessment scale was used to appraise the quality of study for case–control studies and outcomes of interest for cohort studies^13^. The modified Newcastle–Ottawa scale was used for cross-sectional studies^14^. The Cochrane Collaboration’s tool was used to assess the risk of bias for randomized studies, as shown in Supplemental data 1.

### Data abstraction

A structured data collection form was used to derive the following information from each study: title, year the study was conducted, name of the first author, publication year, country where the study was conducted, demographic and characteristic data of CIED devices, and QRS duration before and after implantation.

### Statistical analysis

Analyses were performed using R software, version 3.6.3 (R Foundation for Statistical Computing. Adjusted point estimates from each included study were combined by the generic inverse variance approach of DerSimonian and Laird, which designated the weight of each study based on its variance^15^. Given the possibility of between-study variance, we used a random-effects model rather than a fixed-effect model network meta-analysis model. Bayesian network meta-analysis model was used. To assess the magnitude of heterogeneity, we performed a comparison of the posterior distribution of the estimated heterogeneity variance with its predictive distribution. Surface under cumulative ranking curve was used to rank the treatment for all outcomes^16^.

We evaluated consistency (agreement between direct and indirect evidence) statistically using a design by node splitting test as shown in Supplemental Data 1. This consistency test allowed us to confirm that the selection, or nonselection, of specific comparisons is not related to an actual effect size of that comparison^17,18^.

*Cochrane Handbook for Systematic Reviews of Interventions* was used as reference for risk of bias assessment. Also, Grading of Recommendations Assessment, Development and Evaluations framework was performed to assess if the certainty of information accounted for the network estimates of the main outcomes from individual studies^19^.

We assessed if the primary outcomes, QRS duration changes, remained statistically significant in subgroup analysis based on sample size and study year of individual studies^20^. Brooks–Gelman Rubin diagnostic was performed to assess convergence of models (Supplemental Data 1).

For clinical endpoints, only changes in LVEF were able to be analyzed due to limited availability from included studies. Hence, we also compared LVEF changes before and after pacing device implantation as an exploratory analysis.

Sensitivity analysis was performed by comparing the results between frequentist network meta-analysis approach and Bayesian network meta-analysis approach. Level of study biases was also included in the sensitivity analysis (Supplemental Data 1).

## Results

A total of 707 potentially eligible articles were identified using our search strategy. After the exclusion of duplicate articles, case reports, correspondences, review articles, in vitro studies, pediatric patient population, or animal studies, 34 articles remained for full-length review. There were 20 articles excluded from the full-length review as the QRS duration changes were not reported.

**Figure 1:**
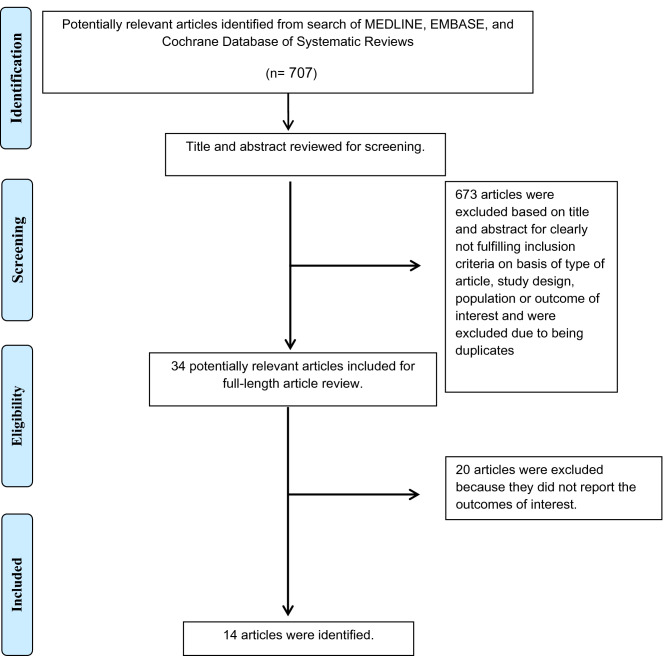
The literature retrieval, review and selection process.

Thus, the final analysis included 14 studies (6 randomized studies and 8 observational studies)^8,21–33^ including 2054 patients. The literature retrieval, review, and selection process are illustrated in Fig. 1. The characteristics and quality assessment of the included studies are presented in Table 1 and Supplemental data 1.

**Table 1:** Study characteristic.

Author	Year	Study type	Population	Pacing indication	RV	CRT	His pacing	LBBP	Outcome
Abdelrahman	2018	Observational study	737	Sinus node dysfunction and AV node dysfunction	Y	N	Y	N	QRS duration
Albertsen	2008	Randomized	50	AV block	Y	Y	N	N	QRS duration
Cai	2019	Observational study	78	Sinus node dysfunction	Y	N	N	Y	QRS duration
Chen	2018	Observational study	40	Sinus node dysfunction and AV node dysfunction	Y	N	N	Y	QRS duration
Upadhyay	2019	Randomized controlled trial	41	CRT indication	N	Y	Y	N	QRS duration
Hou	2019	Observational study	104	Sinus node dysfunction and AV node dysfunction	Y	N	Y	Y	QRS duration
Hua	2020	Observational study	224	Sinus node dysfunction and AV node dysfunction	N	N	Y	Y	QRS duration
Lustgarten	2015	Randomized crossover	34	CRT indication	N	N	Y	Y	QRS duration
Occhetta	2006	Randomized crossover	32	AV node ablation for AF	Y	N	Y	N	QRS duration
Sharma	2014	Observational study	173	Sinus node dysfunction and AV node dysfunction	Y	N	Y	N	QRS duration
Wang	2019	Randomized	131	Sinus node dysfunction and AV node dysfunction	Y	N	N	Y	QRS duration
Wang	2020	Observational study	40	CRT indication	N	Y	N	Y	QRS duration
Wu	2020	Observational study	135	CRT indication	N	Y	Y	Y	QRS duration
Zhang	2020	Randomized	235	Sinus node dysfunction and AV node dysfunction	Y	N	N	Y	QRS duration

**Figure 2:**
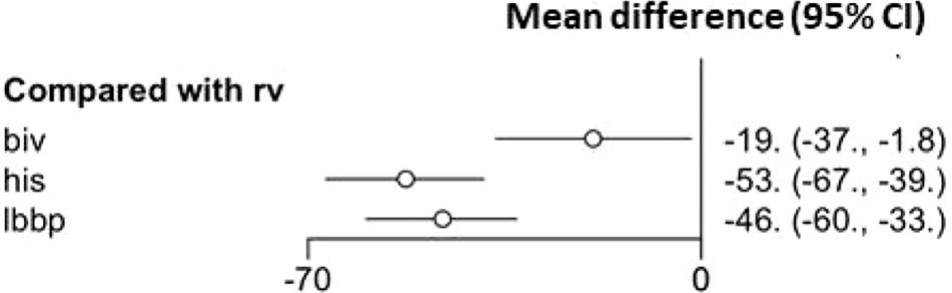
Forrest plot demonstrating relative effect size (QRS duration change pre implantation and post implantation) compared Pacing types with conventional RV apical pacing. Circle data markers represent mean difference of QRS duration between types of pacing compare to RV apical pacing, and horizontal lines represent 95% confident interval (CIs).

**Figure 3:**
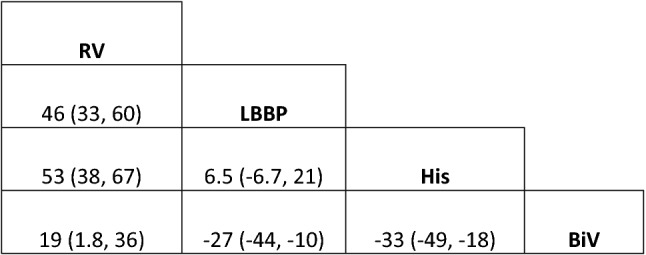
League tables showing the results of the network meta-analyses comparing the QRS duration between RV pacing, Left bundle branch pacing, His bundle pacing and Biventricular pacing. Mean difference between type of pacing and 95% CI. Mean difference less than 1 means the top-left treatment is better, in terms of QRS interval reduction with pacing compared to the baseline.

**Figure 4:**
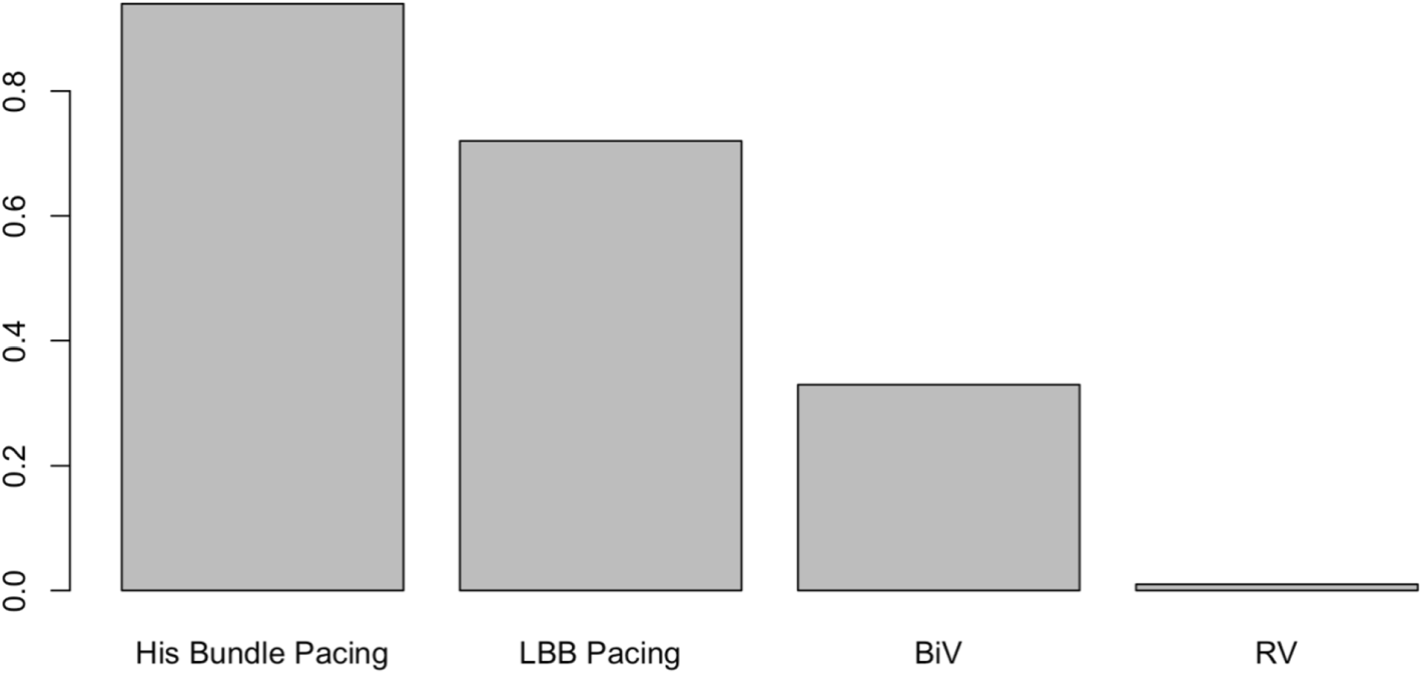
The surface under the cumulative ranking curve (SUCRA) represented overall ranking effect of QRS reduction between pacing types. His bundle pacing showed highest reduction of QRS duration follow by LBB pacing and BiV. Conventional RV apical pacing showed lowest ranking.

The mean study sample size was 185 subjects. For individual implantation, 926 patients were assigned to conventional RV apex implantation, 146 for BiV, 362 for LBBP, and 620 for HBP. When compared to conventional RV pacing, HBP patients had the greatest QRS narrowing with a mean difference of − 53 ms (95% CI − 67, − 39), followed by LBBP with a mean difference of − 46 ms (95% CI − 60, − 33), and BiV with a mean difference of − 19 ms (95% CI − 37, − 1.8) Fig. 2 When compared between LBBP and HBP, showed no statistically significant wider QRS duration in LBBP with mean different 6.5 ms. (95% CI − 6.7, 21). Compared to BiV, both LBBP and HBP patients were significant narrower QRS duration with mean different − 33 ms. (95% CI − 49, − 18) in HBP and − 27 ms. (95%CI − 44, − 10). League table showing pairwise comparison between treatment was showed in Fig. 3. The result of surface under cumulative ranking curve is illustrated in Fig. 4.

We also perform exploratory analysis comparing LVEF change after implantation. There were 6 out of 14 studies with LVEF preimplantation and postimplantation with total population of 465 individuals^8,22,23,25,28,31^. The number of patients implanted with conventional RV apical pacing was 79, patients implanted with BiV was 75, patients implanted with LBBP was 165, and patients implanted with HBP was 146 patients. Post implant follow-up time was up to 12 months. There was no statistically significant difference between types of pacing compared to RV apical pacing. The mean different of BiV compared to RV apical pacing was − 4.9%(95% CI − 21, 11), LBBP was 5.5% (95% CI − 11, 22), and HBP was 3.3% (95%CI − 13, 20). The result of subgroup analysis is shown in Supplemental Data 1.

Sensitivity analysis was performed by comparing the results of network meta-analysis between the Bayesian method and the frequentist method. Categorization of studies according to degree of bias was also used to perform the sensitivity analysis. The results were consistent and showed that HBP and LBBP provided the most change in QRS narrowing (Supplemental Data 1).

Meta-regression to exclude study biases was performed and once again demonstrated the most dramatic decline of QRS duration with HBP and LBBP (Supplemental Data 1).

## Discussion

This study demonstrates that physiologic pacing interventions, both HBP and LBBP, maintain normal physiology of cardiac conduction systems, as shown in the electrocardiogram of patients requiring a permanent pacemaker. Furthermore, these results support the hypothesis that physiologic pacing causes less cardiac dyssynchrony compared to traditional RV apical pacing, consistent with previous studies which showed the HBP technique could improve cardiac function by maintaining myocardial segment electrical activation^34–36^. In patients with preexisting bundle branch block, the long helix His bundle lead may penetrate distally to the level of cardiac conducting system blockage and normalize the QRS complex. While many theories have tried to explain this QRS normalization, functional longitudinal dissociation between bundle branches is believed to be the fundamental physiology of the change^35,37^.

Network meta-analysis was advantageous in that we can indirectly compare head to head of all types of pacing intervention at once, especially to compare with LBBP of which current data remains somewhat limited. Another advantage of network meta-analysis was that we could calculate ranking and hierarchy of interventions showing that physiologic pacing was the highest rank of all interventions to yield the narrowest QRS duration.

QRS duration is a powerful marker for cardiac dyssynchrony. The prolongation of QRS complex to 120 ms or longer is associated with more advanced myocardial disease, poorer prognosis, and higher all-cause mortality compared to a normal QRS complex duration^38^. In patients with an LVEF less than 30%, QRS prolongation is associated with increased mortality and sudden cardiac death. Furthermore, in patients with an LVEF of 30–40%, QRS prolongation is associated with increased mortality^39^. QRS duration is the major determinant for CRT according to current guidelines^40^. The results from our study showed a significantly narrower QRS duration in patients with HBP and LBBP compared to BiV; thus, physiologic pacing can be translated into better cardiac performance by restoring normal interventricular electrical activation patterns.

The current guidelines recommend BiV-based interventions in patients with chronic atrial fibrillation and heart failure who underwent atrial ventricular node ablation due to inadequate control of heart rate by medications^41^. However, several studies have shown no benefit in patients with previously narrow QRS complex, which could be explained by remaining electrical dyssynchrony after BiV^42,43^.

Surprisingly, our exploratory analysis suggested that physiologic pacing and BiV did not yield better echocardiographic outcomes than conventional RV pacing. There were major concerns about this result. First, the methods used to measure results were found to be vastly different. Some studies used 3-dimensional echocardiography measurement; some used 2-dimensional biplane method. Second, there was also heterogeneity in follow-up time. The longest follow-up time was 12 months. The shortest follow-up time was immediately after the procedure. This might have affected the results of the analysis. Third, only six studies were available for retrieving this relevant information. Hence, underpower could be an issue leading to underestimation in LVEF changes from these devices.

The implantation of HBP comprises delivery of the RV lead into the area of His Purkinje system with a 3830 pacing lead and C315 His non deflectable sheath^44^. Once the area of His signal is obtained, the lead is then screwed into myocardium^44,45^. The success rate of this procedure has been reported as up to 92% in experienced centers^46^, and was found not to be different from the success rate of BiV^47^. The issues with HBP that concern most operators are long-term lead stability and ventricular capture threshold. Primarily, the pacing output threshold, the least electrical energy delivered that triggers electrical depolarization, would increase over time; 6.7% of patients required lead revision over 5 years of follow-up^46,48^.

Another unresolved issue with HBP interventions is increased battery drainages secondary to higher ventricular capture thresholds^45^. The implantation of LBBP is similar to the HBP implantation procedure, with the same type of lead and sheath, as well as methods of delivering the lead, used in both implantation processes. The difference between LBBP and HBP procedures is that once the His bundle electrogram is obtained, the tip of pacing lead is moved 1.5–2 cm toward the ventricular apex on the right anterior oblique fluoroscopic projection, and pace-mapping is performed to secure the lead in the ideal position^49^. The successful LBBP would result in right bundle branch morphology with a QRS duration of less than 130 ms. The issues with HBP (increased pacing and sensing threshold) do not occur in LBBP^26,50^.

Current evidence has pointed toward higher success rates and lower pacing thresholds in LBBP compared to HBP^51,52^. Although both techniques appear to be relatively safe in short-term follow-up and, theoretically, advantageous over conventional RV pacing, many questions remain to be answered in the clinical setting. For example, the mortality benefit and rate of heart failure hospitalization remain unknown for both procedures. Nevertheless, the results of the present study provided additional evidence to support that physiologic pacing, both HBP and LBBP, is associated with narrower QRS duration compared to conventional RV pacing. Narrowing of QRS duration is related to a lower electromechanical dyssynchrony, and thus, HBP and LBBP may confer lower incidence of adverse cardiac events from pacing-induced cardiomyopathy.

### Limitations

QRS duration was the only parameter analyzed in our study. Other markers of synchronous contraction were not specified in included studies, precluding further analysis. Nevertheless, many studies have suggested QRS duration is the best surrogate marker for cardiac synchronicity^38,53^. Since physiologic pacing, particularly LBBP, has been in the early phase of trials, the lack of clinical endpoints is inevitable. Further studies are required to establish health impacts among patients receiving either HBP or LBBP. Secondly, half of the studies we included in our analysis were observational studies. Thus, residual biases cannot be completely excluded. Despite this caveat, the Newcastle–Ottawa scale criteria were adopted to stratify biases risks as well as study qualities, suggesting robust analysis. Thirdly, the total number of patients in our study was small, possibly leading to an underestimation of the actual effects. Lastly, almost all the studies were done in centers with expertise in physiologic pacing. Therefore, the success rates and results might not be applicable to general or low volume clinical settings. Despite these limitations, this study is the first network meta-analysis to provide the most updated comparison of the performance of physiologic pacing compared to conventional pacing.

## Conclusion

Our study has demonstrated that HBP and LBBP result in narrower QRS duration compared to BiV and conventional RV apical pacing. Although clinical outcomes were not studied, these results suggest the advantage of near-normal ventricular depolarization in physiologic pacing interventions. Further analysis should be done to demonstrate the clinical benefit of physiologic pacing. We believe that new battery systems, delivery tools, and lead technologies being developed in the near future will be the key to improved feasibility and success rate of physiologic pacing interventions.

## Supplementary information

Supplementary Information.

## Abbreviations

BiV: Biventricular pacing
CRT: Cardiac resynchronization therapy
CIED: Cardiac implantable electronic device
HBP: His bundle pacing
LBBP: Left bundle branch pacing
LV: Left ventricle
LVEF: Left ventricular ejection fraction
PCM: Pacemaker induced cardiomyopathy
RV: Right ventricle
