# Supplementary material for: A network meta-analysis and systematic review of change in QRS duration after left bundle branch pacing, His bundle pacing, biventricular pacing, or right ventricular pacing in patients requiring permanent pacemaker
Source: Sci Rep. 2021 Jun 9;11:12200. doi: 10.1038/s41598-021-91610-8 (PMC8190182; doi:10.1038/s41598-021-91610-8)
Supplement: Supplementary file 1 — Supplementary Information. [file 41598_2021_91610_MOESM1_ESM.docx]

**Supplemental data 1**

Supplementary to: **A Network Meta-analysis and Systematic Review of Change in QRS Duration After Left Bundle Branch Pacing, His Bundle Pacing, Biventricular Pacing, or Right Ventricular Pacing in Patients Requiring Permanent Pacemaker**

Author: Nithi Tokavanich MD , Narut Prasitlumkum MD, Wimwipa Mongkonsritragoon MD , Wisit Cheungpasitporn, MD, Charat Thongprayoon, MD, Saraschandra Vallabhajosyula, MD MSc, Ronpichai Chokesuwattanaskul MD^1^*

This supplementary data provided the result of subgroup analysis, authors’ search strategy and result, evaluation of consistency by Node-splitting, Gelman-Rubin diagnosis plot and sensitivity analysis.

**Table of content Page**

Search strategy 3-4

Node split (QRS outcome) 5

Gelman plot (QRS outcome) 6

Network meta-analysis of low risk and high risk bias study 7-8

Bias assessment 9-12

Meta regression (QRS outcome) 13

Sensitivity analysis 14

Result of subgroup analysis 15-17

**Part 1 search strategy**


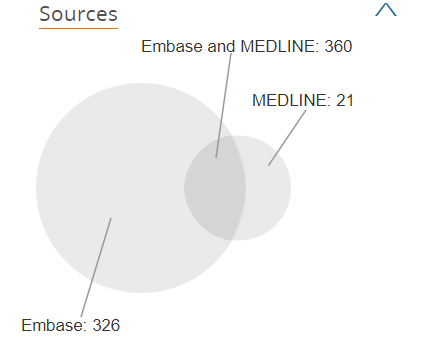


Session Results

| Number | Query | Results |
| --- | --- | --- |
| 19 | #10 OR #11 OR #12 OR #13 OR #14 OR #17 OR #18 | 707 |
| 18 | #3 AND #4 | 0 |
| 17 | #3 AND #15 | 19 |
| 16 | #5 AND #15 | 6 |
| 15 | #6 OR #8 | 31764 |
| 14 | #3 AND #9 | 12 |
| 13 | #5 AND #9 | 0 |
| 12 | #6 AND #9 | 309 |
| 11 | #7 AND #9 | 0 |
| 10 | #8 AND #9 | 641 |
| 9 | 'right ventricular pacing' | 2145 |
| 8 | 'cardiac resynchronization therapy' | 31261 |
| 7 | 'chronic resynchornization' | 0 |
| 6 | 'biventricular pacing' | 2983 |
| 5 | left bundle branch block pacing' OR 'lbbb pacing | 7 |
| 4 | left AND bundle AND branch AND block AND pacing | 3670 |
| 3 | 'his pacing' | 76 |

**Evaluation of consistency of Network Meta-analysis of QRS duration**


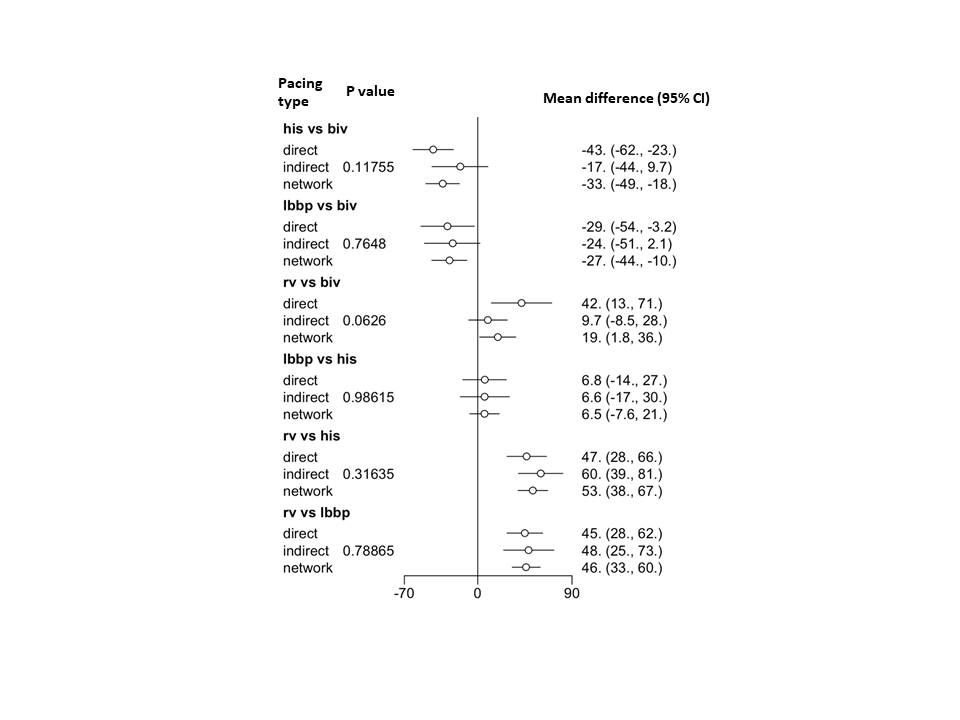


Node splitting analysis compares between pacing types (HBP and LBBP and RV apical pacing)

Circle data markers represent mean difference of QRS duration between types of pacing compare to RV apical pacing, and horizontal lines represent 95% confident interval (CIs).


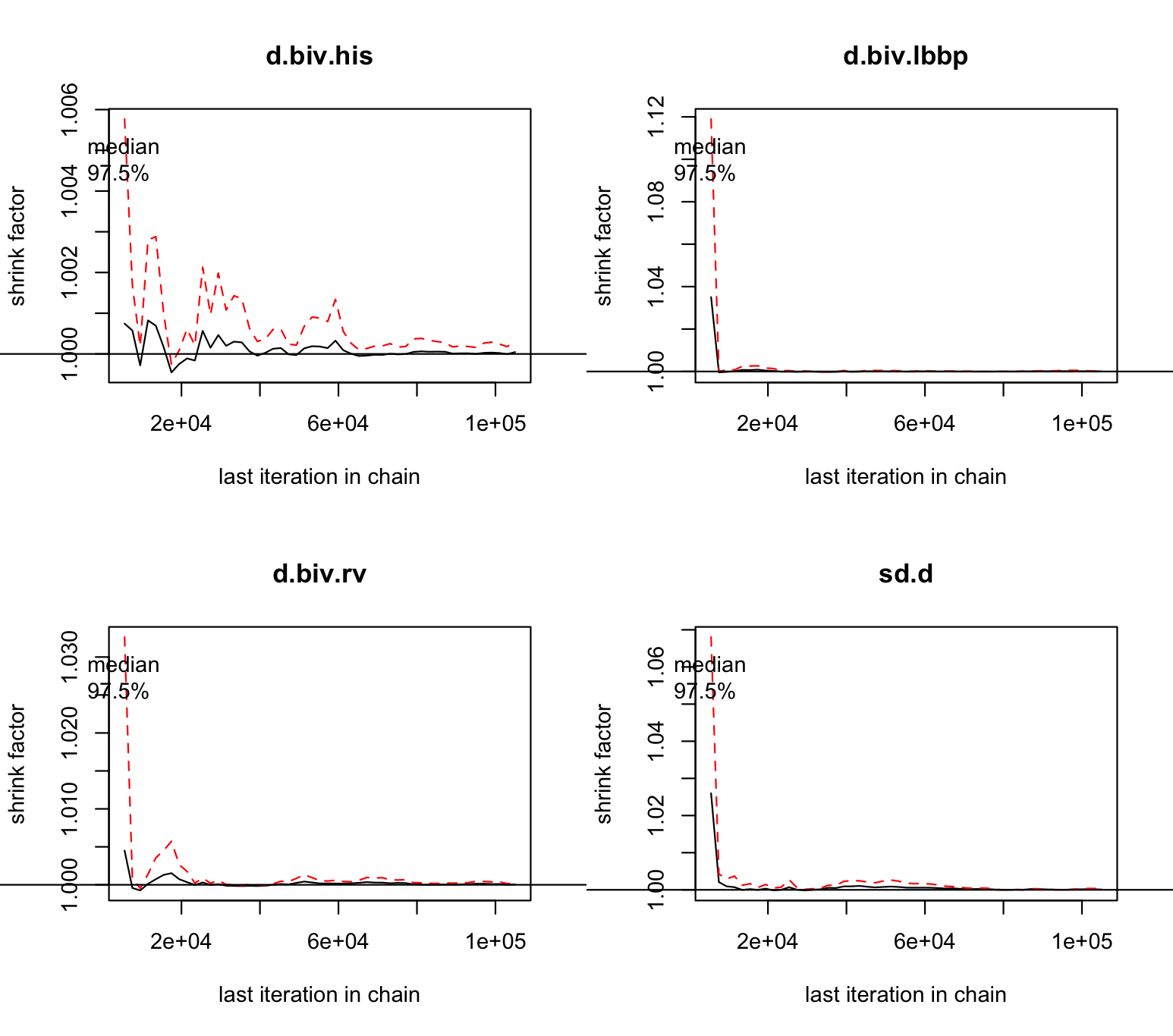


Gelman-Rubin plot of QRS duration outcome

Gelman Rubin diagnostic was performed to assess convergence of models

**
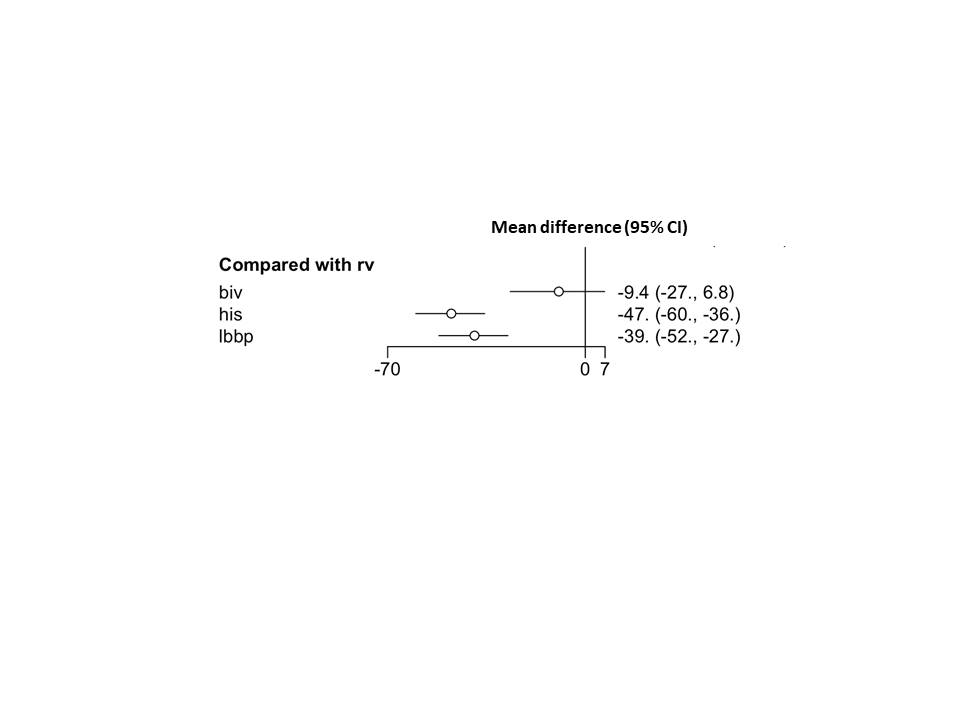
**

Network Meta-analysis of Low risk of Biases studies of QRS duration

Circle data markers represent mean difference of QRS duration between types of pacing compare to RV apical pacing, and horizontal lines represent 95% confident interval (CIs)

**
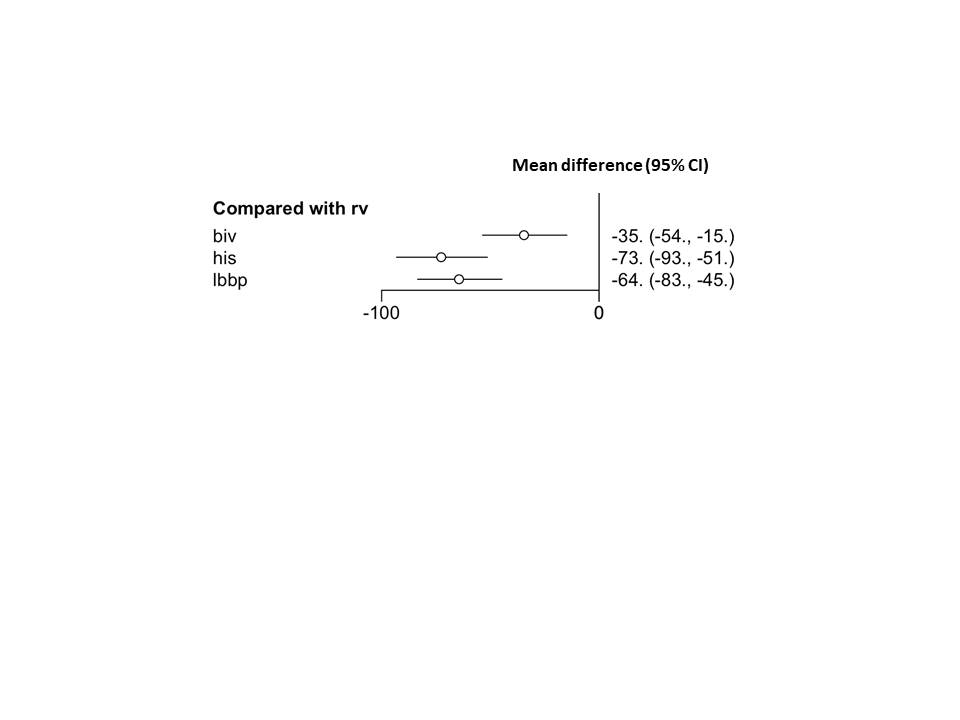
**

Network Meta-analysis of High risk of Biases studies of QRS duration

Circle data markers represent mean difference of QRS duration between types of pacing compare to RV apical pacing, and horizontal lines represent 95% confident interval (CIs)

**Cochrane risk of bias assessment**


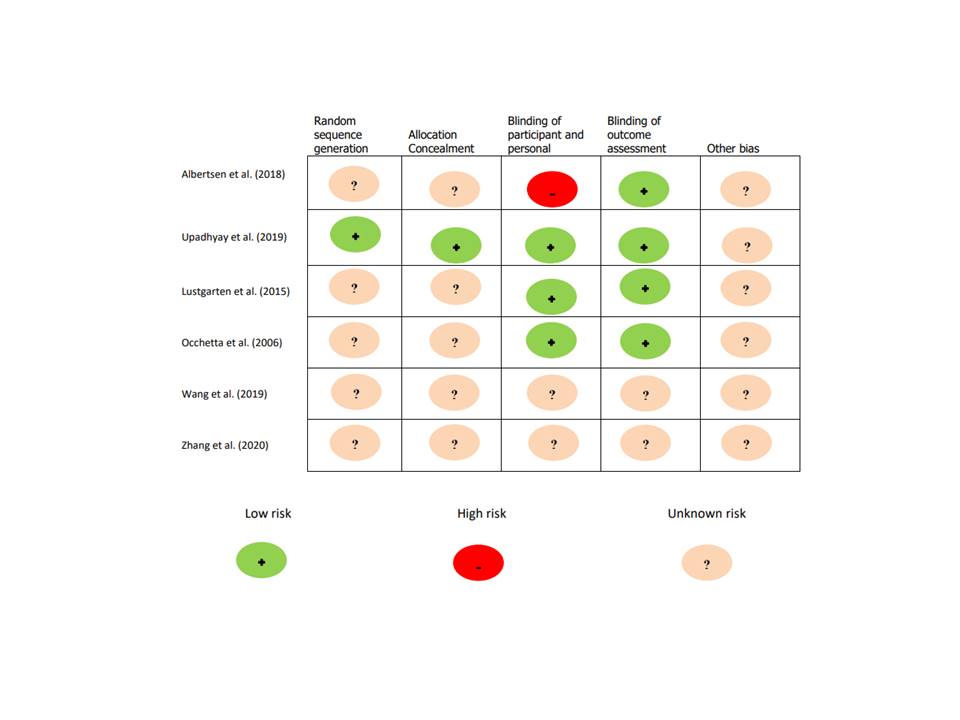


| Study | Selection | | | | | Comparability (Confounding) | Outcome | | | Total |
| --- | --- | --- | --- | --- | --- | --- | --- | --- | --- | --- |
|  | Representative of exposed cohort | Selection of the non-exposed cohort | Ascertainment of exposure | Endpoint not presenting at start |  | | Assessment of Outcome | Follow-up duration | Adequacy follow-up |  |
| Abdelrahman | * |  | * | * | * | |  | * | * | 6 |
| Cai | * | * | * | * | ** | |  | * | * | 8 |
| Chen | * | * | * | * |  | | * | * | * | 7 |
| Hou | * | * | * | * | ** | |  | * | * | 8 |
| Hua | * | * | * | * | ** | |  | * | * | 8 |
| Wang | * | * | * | * |  | |  | * | * | 6 |
| Wu | * | * | * | * | ** | | * | * | * | 9 |

**Newcastle-Ottawa quality assessment scale of included studies in meta-analysis** (cohort)

**Newcastle-Ottawa quality assessment scale of included studies in meta-analysis** (case-control)

| Study | Selection | | | | Comparability (Confounding) | Exposure | | | Total | |
| --- | --- | --- | --- | --- | --- | --- | --- | --- | --- | --- |
|  | Adequacy of definition | Representativeness of the cases | Selection of controls | Definition of controls |  | Assessment of Exposure | Same methods for ascertainment | Nonresponse rate |  |  |
| Sharma | * | * | * | * | * | * | * |  | 7 |  |


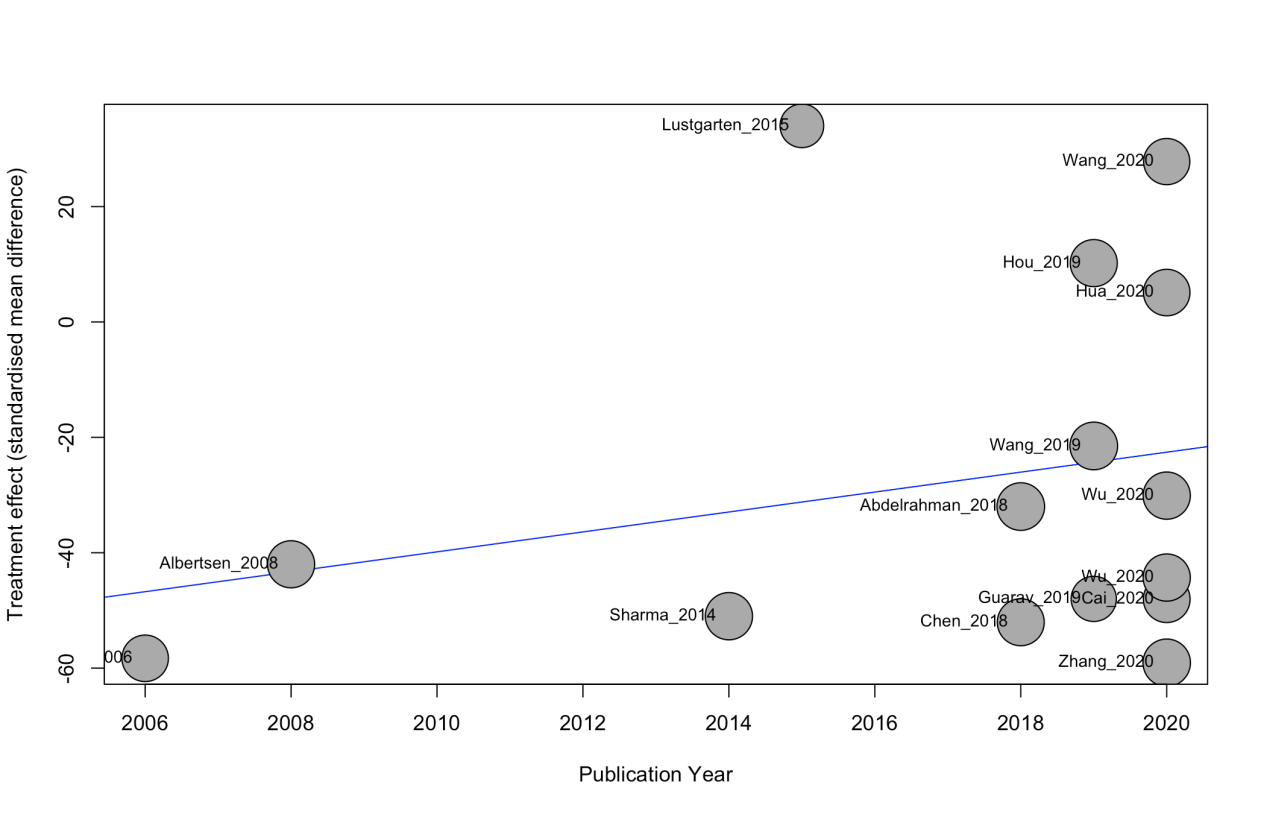


Network meta-regression according to the publication year does not affect treatment effect with P value 0.37

Circle data markers represent study, Y plain represented treatment effect (mean different of QRS duration), X plain represented publication year.

**Sensitivity analysis with Frequentist Network Meta-analysis**

**P score ranking**: HBP yielded the highest ranking in reduction of QRS follow by LBBP and BiV

**Result of Subgroup analysis of LVEF pre implantation and post implantation**

**
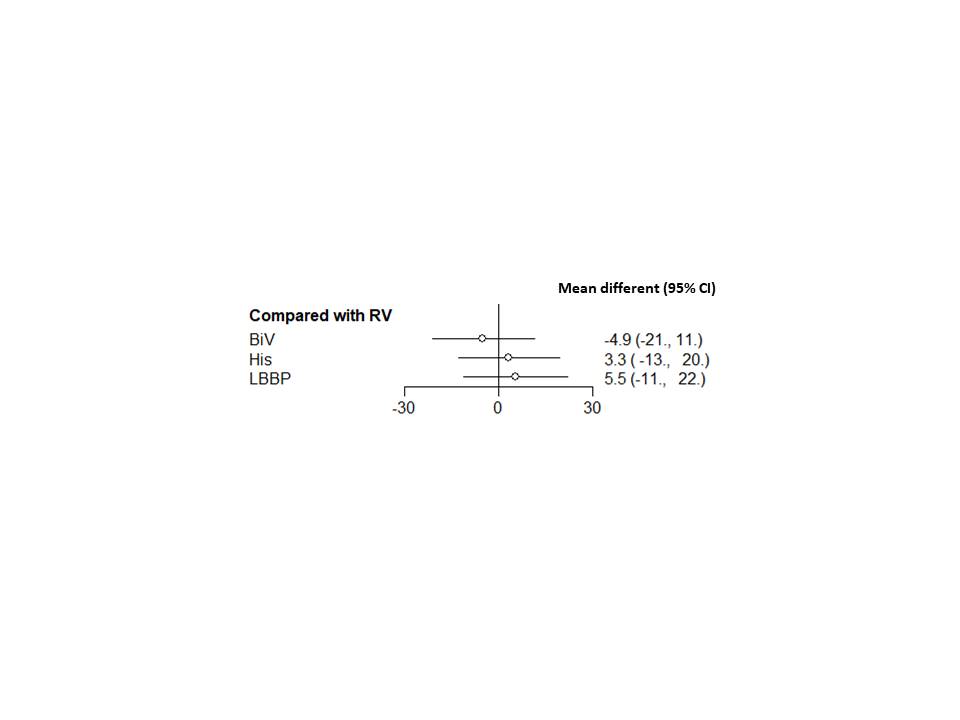
**

Forrest plot comparing pre implantation LVEF and post implantation LVEF

Circle data markers represent mean difference of LVEF between types of pacing compare to RV apical pacing, and horizontal lines represent 95% confident interval (CIs)


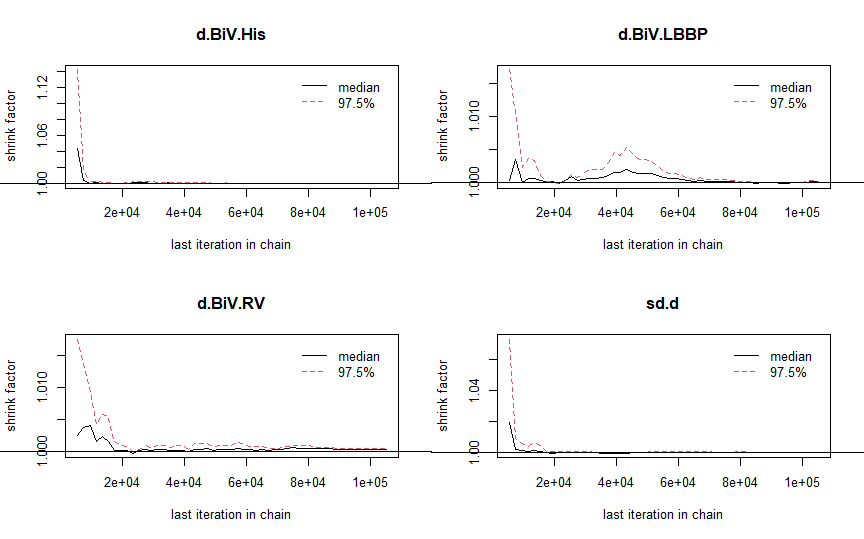


Gelman-Rubin plot of pre implantation and post implantation LVEF

Gelman Rubin diagnostic was performed to assess convergence of models.


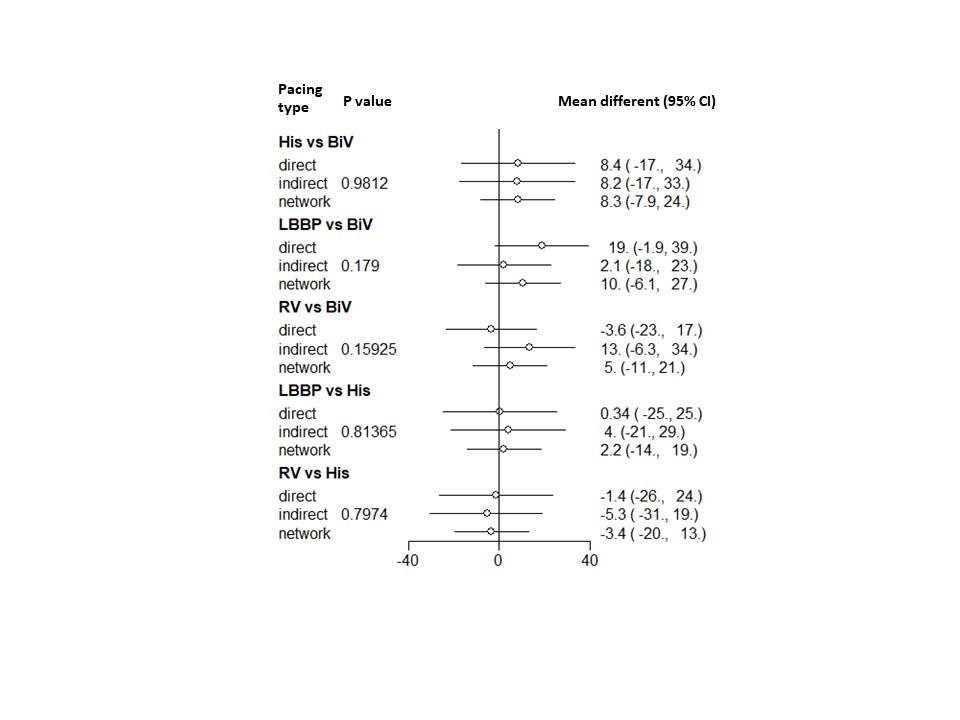


Node splitting analysis

Circle data markers represent mean difference of QRS duration between types of pacing compare to RV apical pacing, and horizontal lines represent 95% confident interval (CIs).
